# Oxyntomodulin increases intrinsic heart rate through the glucagon receptor

**DOI:** 10.1002/phy2.112

**Published:** 2013-10-20

**Authors:** Auyon Mukharji, Daniel J Drucker, Maureen J Charron, Steven J Swoap

**Affiliations:** 1 Department of Biology, Williams CollegeWilliamstown, Massachusetts; 2 Department of Medicine, Samuel Lunenfeld Research Institute, Mt. Sinai Hospital, University of TorontoToronto, Canada; 3 Departments of Biochemistry, Medicine, and Ob/Gyn and Women's Health, Albert Einstein College of MedicineNew York, New York

**Keywords:** Beta block, GLP-1, OXM, sympathetic nervous system

## Abstract

Two hormones from the gastrointestinal tract, glucagon and oxyntomodulin (OXM), vigorously elevate the intrinsic heart rate (I_HR_) of mice. We have previously shown that OXM influences murine heart rate (HR) independent of the glucagon-like peptide 1 (GLP-1) receptor. Here, we demonstrate using radiotelemetry in mice deficient in the glucagon receptor (*Gcgr* −/−) that both OXM and glucagon require the glucagon receptor for their chronotropic effects on the heart. Furthermore, we found that other hormones associated with hunger and satiety (ghrelin, leptin, and PYY_3-36_) had no effect on I_HR_, while cholecystokinin moderately elevated the I_HR_. Finally, the resting HR of *Gcgr* −/− mice was higher than in control mice (*Gcgr* +/+ and *Gcgr* +/−) at thermal neutral temperature (30°C). Using atropine, we demonstrated that *Gcgr* −/− mice have diminished parasympathetic (PNS) influence of the heart at this temperature. *Gcgr* −/− mice displayed a normal bradycardia as compared to controls in response to administration of either methacholine (to activate the muscarinic acetylcholine receptor) or methoxamine (to activate the baroreflex through agonism of the α1 adrenergic receptor agonist) suggesting that vagal pathways are intact in the *Gcgr* −/− mice. As OXM is an agonist of the GLP-1 receptor and Gcgr with antidiabetic activity, we suggest OXM may be an alternative to glucagon in the treatment of overdose of beta-blockers to elevate HR in clinical conditions.

## Introduction

The gastrointestinal tract is the body's largest endocrine organ, and releases more than 20 different regulatory peptide hormones that act on a number of tissues, including exocrine glands, cardiac muscle, smooth muscle, and the peripheral nervous system (Murphy and Bloom 2006; Drucker 2007). The changes in the circulating levels of these gut hormones and the interactions between them are known to modulate numerous physiological functions. The most well studied of these functions are hunger and satiety (Cummings and Overduin 2007; Chaudhri et al. 2008; Karra and Batterham 2010). Because some gut hormones could be used as a therapeutic to either increase hunger, as in treatment of cachexia, or decrease hunger, as in treatment of obesity, it is important to identify other physiological systems that are affected by hunger-modulating hormones. In this study, we examined the effects of some of these hormones on the intrinsic heart rate (I_HR_) of mice.

Heart rate (HR) is governed by the interactions between the intrinsic HR and the autonomic nervous system, which is comprised of the sympathetic nervous system (SNS) and the parasympathetic nervous system (PNS). The I_HR_ is the rate of contractions occurring under conditions of no nervous input on cardiac tissue. Known modulators of I_HR_, such as adenosine and thyroid hormone, are known to function by affecting specific channels or currents that determine the potential of the membrane (Belardinelli et al. 1995; Foley et al. 2001; De Angelis et al. 2004; Takayama et al. 2005). For example, the primary action of adenosine in sinoatrial node cells is the direct cAMP-independent activation of the inwardly rectifying potassium ion current, which slows the I_HR_ (Belardinelli et al. 1995).

Glucagon, a 29-amino acid pancreatic hormone derived from processing of proglucagon in the alpha cells of the islets of Langerhans, has long been known to elevate I_HR_ (Steiner et al. 1969; Stuesse et al. 1982), which is also true in mice (Sowden et al. 2007). Because of its direct impact on the heart to increase HR independent of the SNS, glucagon is often used a first line of defense clinically to elevate HR in cases of beta-blocker poisoning (Newton et al. 2002; Watson et al. 2005; Shepard 2006; Kerns 2007). Glucagon has a great number of effects throughout the body of which most are mediated via a specific glucagon receptor, Gcgr, expressed in numerous tissues including the heart (Burcelin et al. 1995; Hansen et al. 1995; Dunphy et al. 1998; Mayo et al. 2003; Habegger et al. 2010). When glucagon binds its receptor, adenylate cyclase is activated, increasing cAMP levels and activating a cAMP-dependent protein kinase (Macneil et al. 1994). While glucagon is probably best known for its effects on the liver in glucose homeostasis, the hormone also has a number of extrahepatic effects, including positive inotropic and chronotropic effects in the heart, increased lipolysis in adipose tissue, action as a satiety factor in the central nervous system, regulatory effects on glomerular filtration rate, and intraislet regulation of insulin, glucagon, and somatostatin secretion (Habegger et al. 2010; Heppner et al. 2010; Vuguin and Charron 2011).

Oxyntomodulin (OXM) is a 37-amino acid peptide containing the entire 29-amino acid sequence of glucagon followed by an eight amino acid carboxy-terminal extension and is released postprandially from the intestinal L-cells (Druce et al. 2004; Druce and Ghatei 2006; Pocai 2012). OXM dose-dependently inhibits food intake under normal and fasting conditions without delaying gastric emptying (Cohen et al. 2003; Dakin et al. 2004; Sowden et al. 2007). In addition to its effects on food intake, OXM strongly elevates HR without effecting blood pressure and lowers core body temperature (Sowden et al. 2007). OXM has a weak affinity for the Gcgr and the glucagon-like peptide 1 receptor (GLP-1R) (Dakin et al. 2001; Baggio et al. 2004). It is through the GLP-1R that OXM exerts its effects on satiety, elevates insulin secretion from β cells and lowers body temperature (Sowden et al. 2007; Maida et al. 2008). However, OXM accelerates murine HR independent of the autonomic nervous system through a GLP-1R-independent mechanism (Sowden et al. 2007).

This study was designed to (1) examine the effects of other gut hormones on the I_HR_ of mice, and (2) test the hypothesis that the cardiac effects of OXM are mediated through the Gcgr. We show here that the effect of OXM on I_HR_ requires 10× the dose of glucagon and is dependent upon the action of the Gcgr. We suggest OXM be considered for study in place of or in addition to the use of glucagon to elevate HR in human patients that have cardiotoxicity associated with an overdose of beta-blockers or calcium channel blockers.

## Materials and Methods

### Animals

Six adult female C57Bl/6J mice, weighing ∼25 g, were purchased from Jackson Labs (Bar Harbor, ME). *Gcgr* −/− mice used in this study were originally described by Gelling et al. (2003). Mice were housed individually at 30°C in a 12:12 h light–dark cycle and fed ad libitum on the Harlan Teklad (Madison, WI) mouse/rat laboratory diet. All procedures and experimental protocols were approved by the Williams College Animal Care and Use Committee.

### Genotyping

The offsprings from four *Gcgr +/*− breeding pairs were genotyped. DNA was purified using from tail snips using a QIAamp Tissue Kit (Qiagen, Germantown, MD). The polymerase chain reaction (PCR) was run using a ReadyMix (Sigma, St. Louis, MO). The primers used are listed in Table 1.

**Table 1:** Primers for genotyping *Gcgr* +/− progeny

Gcgr forward primer	TCCCAATGTCAGTTGGATGA
Gcgr reverse primer	AAGGTGAGGCATGAGTGGAG
Neomycin cassette forward primer	GTCTTGTCGATCAGGATGATCTG

### Reagents

Porcine OXM was obtained from California Peptide Research Inc. (Napa, CA). Glucagon, metoprolol, methoxamine, methacholine, and atropine were obtained from Sigma.

### Implantation of ECG telemeters

Mice were anesthetized with 2–3% isofluorane in an oxygen stream, and then maintained at ∼2% isofluorane. The mice were kept on a heating pad throughout the implantation of the electrocardiogram (ECG) telemeters (ETAF20, weight 1.6 g with a volume of 1.1 cc, Data Sciences International, St. Paul, MN) in the abdominal cavity with subcutaneous placement of the leads. Mice were maintained on a heating pad for at least 24 h after surgery, and were then housed at 30°C for 10 days in order to ensure adequate recovery.

### Cardiovascular, temperature, and activity data collection

Data from the ECG telemeters were sampled at 2000 Hz. Data were collected for 5 sec, once per minute. Data were collected for 12 h of the dark phase and 11 h of the light phase as the final hour of the light phase was reserved for animal care. For injections, data were taken from 30 min before the injection until 120 min afterward. Data were binned in 3-min increments for HR and analysis.

### Experimental protocols

For analysis of the effects of meal-related peptides on I_HR_, an autonomic block was induced in six C57BL/6J mice using an intraperitoneal injection of metoprolol (12 mg/kg) and atropine (5 mg/kg) 3 h before the onset of the dark phase. This injection was then followed 25 min later by an intraperitoneal injection of vehicle (saline), OXM (0.3, 1.5, and 15 μg), glucagon (0.3, 1.5, and 15 μg), ghrelin (100 μg), PYY_3-36_ (100 μg), leptin (33 μg), or cholecystokinin (CCK – 10 μg). These doses were chosen using previous publications as a guide (Steiner et al. 1969; Stuesse et al. 1982; Kurosawa et al. 2001; Gluck et al. 2006; Sowden et al. 2007; Parkinson et al. 2008). Mice were randomized through all groups such that each mouse received each condition. A minimum of 3 days transpired before each measurement of I_HR_. The effect of OXM or glucagon on I_HR_ in *Gcgr* −/− (*n* = 9, five males and four females) and control (*n* = 12, six males and six females) mice was measured at one dose (15 and 1.5 μg, respectively). Mice were randomized for each condition (OXM, glucagon, saline) such that each mouse received each of the three conditions. For analysis of PNS and SNS input to HR control, the same mice used in the previous experiment were used again. Experiments were initiated ∼3 h before the onset of the dark phase. Baseline HR was calculated for 30 min prior to an injection of either metoprolol (12 mg/kg), to block SNS input to the heart, or atropine (5 mg/kg), to block PNS input to the heart. The average HR was calculated over a 10 min duration, beginning 20 min after the injection to 30 min after the injection. In those experiments that utilized methacholine (0.15 mg/kg) or methoxamine (6 μg/kg), data were sampled for 30 min before injection of either of these compounds and continued sampling for 160 min.

### Statistics

All results are reported as means ± SE. The effect of genotype on dark and light phase physiological variables was statistically assessed using non-paired *t*-tests. The effect of numerous peptides and the different doses was assessed using a repeated measures analysis of variance (ANOVA) (with comparison to the 3 min prior to injection the peptide). To determine the effect of either atropine or metoprolol on resting HR, a paired *t*-test was used for each compound in each mouse genotype. Significance levels of *P* < 0.05 were accepted.

## Results

### Only a subset of satiation-related peptides influence murine I_HR_

**Figure 1:**
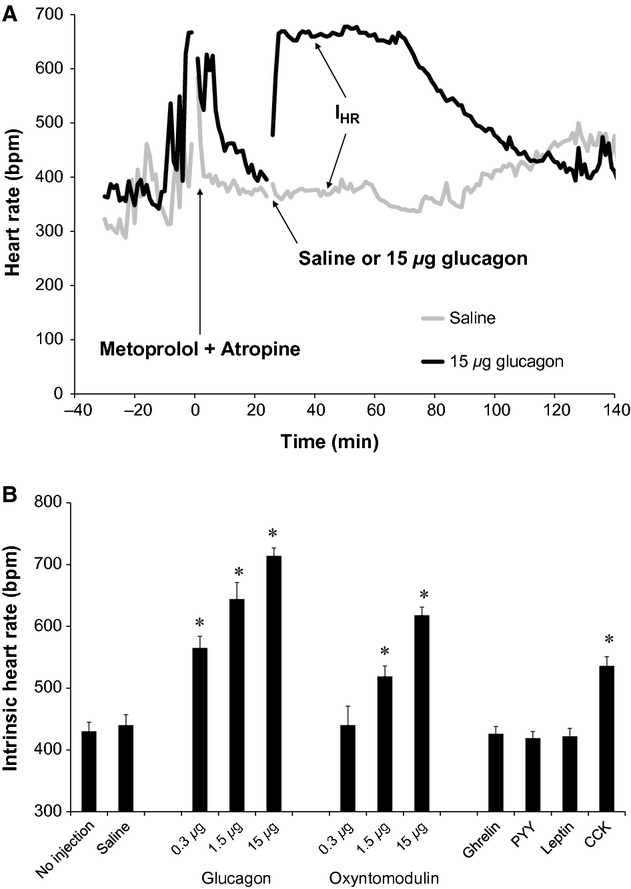
Glucagon and oxyntomodulin (OXM) robustly elevate the I_HR_ of C57Bl/6J mice. (A) A typical female mouse implanted with an ECG telemeter was injected with a cocktail of metoprolol and atropine at time “0”, and 25 min later received an intraperitoneal injection of either saline or 15 μg of glucagon. As is apparent in the saline injection, animal handling has no effect on HR as the autonomic nervous system is blocked. I_HR_ measurements were made in this study 15 min after injection of the compound or vehicle. (B) I_HR_ was measured in female C57Bl/6J mice in response to saline, glucagon (one of three doses), OXM (one of three doses), ghrelin (100 μg), PYY_3-36_ (100 μg), Leptin (33 μg), CCK (10 μg), or with no second injection. While ghrelin, PYY_3-36_, and leptin had no significant effect on I_HR_, CCK moderately elevated I_HR_. Both glucagon and OXM increased I_HR_, with 1.5 μg of glucagon generating a similar response to 15 μg of OXM. *P < 0.05 versus saline.

As OXM and glucagon both elevate I_HR_ in mice (Steiner et al. 1969; Stuesse et al. 1982; Sowden et al. 2007), other peptides known to influence satiation were tested in mice to assess their effect on I_HR_. To measure I_HR_, C57Bl/6J mice were implanted with a telemeter to detect HR. After recovery from the surgery, mice were administered a cocktail of metoprolol and atropine. Mice were then given a second injection of a peptide or vehicle 25 min later. Measurements for I_HR_ were obtained from tracings 15 min after injection of saline (40 min after injection of the cocktail, as shown on Fig. 1A). Figure 1A shows a typical HR response to either saline or 15 μg of glucagon after the autonomic block induced by metoprolol and atropine. As Figure 1B shows, three of the tested hormones, leptin, PYY_3-36_, and ghrelin, had no effect on I_HR_, even at high doses (33, 100, and 100 μg, respectively). The most potent peptide tested was glucagon with a significant elevation in I_HR_ at the lowest dose tested (0.3 μg). OXM also increased I_HR_ with 1.5 μg and 15 μg, as did CCK at a dose of 10 μg.

### *Gcgr* −/− mice exhibited elevated HRs at warm ambient temperatures

To determine whether the Gcgr mediates the action of OXM and glucagon on I_HR_, mice deficient in Gcgr were examined. *Gcgr* +/+, +/−, and −/− mice were implanted with telemeters to detect HR, core body temperature (*T*_b_), and general cage activity. Measurements of these variables were made in the dark phase and light phase, while the mice were housed at an ambient temperature (*T*_a_) of 30ºC (Table 2). Data analysis showed no difference between the *Gcgr* +/+ mice and *Gcgr* +/− mice in any measurement, therefore data from these mice were combined as the “control” group. Control and *Gcgr* −/− mice displayed normal circadian variation, with elevated HR, *T*_b_, and cage activity in the dark phase relative to the light phase (Table 2). While core *T*_b_ and general cage activity were not different between the two groups of mice, *Gcgr* −/− mice had a significantly higher HR than control mice during the light phase (Table 2).

**Table 2:** Physiological variables of control and *Gcgr* −/− mice

	Control mice	*Gcgr* −/− mice
Dark phase heart rate (bpm)	426 ± 18*	429 ± 16**
Light phase heart rate (bpm)	322 ± 7	357 ± 9*
Dark phase core *T*_b_ (°C)	37.5 ± 0.2*	37.2 ± 0.1**
Light phase core *T*_b_ (°C)	35.9 ± 0.2	36.1 ± 0.1
Dark phase activity (A.U.)	12.0 ± 4.0*	12.8 ± 3.0**
Light phase activity (A.U.)	1.3 ± 0.4	3.5 ± 0.7

A.U. = arbitrary units.

* *P* < 0.05 versus light phase for control mice.

** *P* < 0.05 versus light phase for *Gcgr* −/− mice.

### OXM and glucagon elevate I_HR_ via the Gcgr

**Figure 2:**
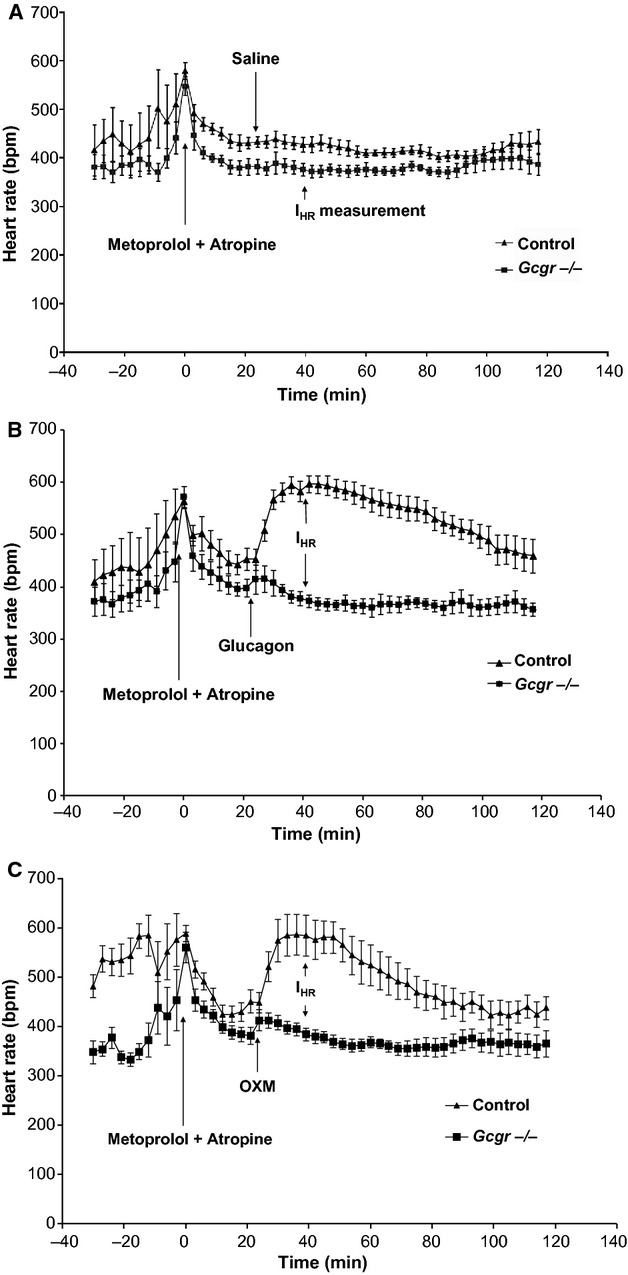
OXM increases I_HR_ through the Gcgr. Control mice and *Gcgr* −/− mice were implanted with ECG telemeters for measurement of heart rate. The mice received a cocktail of metoprolol and atropine at time “0” to block autonomic input to the heart. 25 min after metoprolol/atropine, mice received an intraperitoneal injection of either saline (A), 1.5 μg glucagon (B), or 15 μg OXM (C). Both glucagon and OXM elevated HR significantly in control mice, but neither of these peptides elevated HR in *Gcgr* −/− mice, demonstrating the requirement of the Gcgr for this effect.

The I_HR_ of control and *Gcgr* −/− male and female mice was next assessed. Within either genotype, no difference was found between genders. However, the I_HR_ of control mice was significantly higher (Fig. 2A) than that of *Gcgr* −/− mice (430 ± 12 bpm vs. 382 ± 17 bpm, respectively). To examine whether glucagon and OXM elevate I_HR_ via the Gcgr, both groups of mice received the metoprolol/atropine cocktail, followed 25 min later by a second intraperitoneal injection of either OXM (15 μg), or glucagon (1.5 μg). Glucagon vigorously increased the I_HR_ (the time of measurement of I_HR_ is shown in Fig. 2B) of control mice to 586 ± 42 bpm. As expected, glucagon did not alter the I_HR_ of *Gcgr* −/− mice (394 ± 14 bpm). Like glucagon, OXM robustly elevated I_HR_ in control mice to 595 ± 16 bpm (Fig. 2C). However, OXM had no effect on the I_HR_ of *Gcgr* −/− mice, as their I_HR_ remained at 381 ± 12 bpm (Fig. 2C).

### Inappropriately low PNS to the heart in *Gcgr* −/− mice

**Figure 3:**
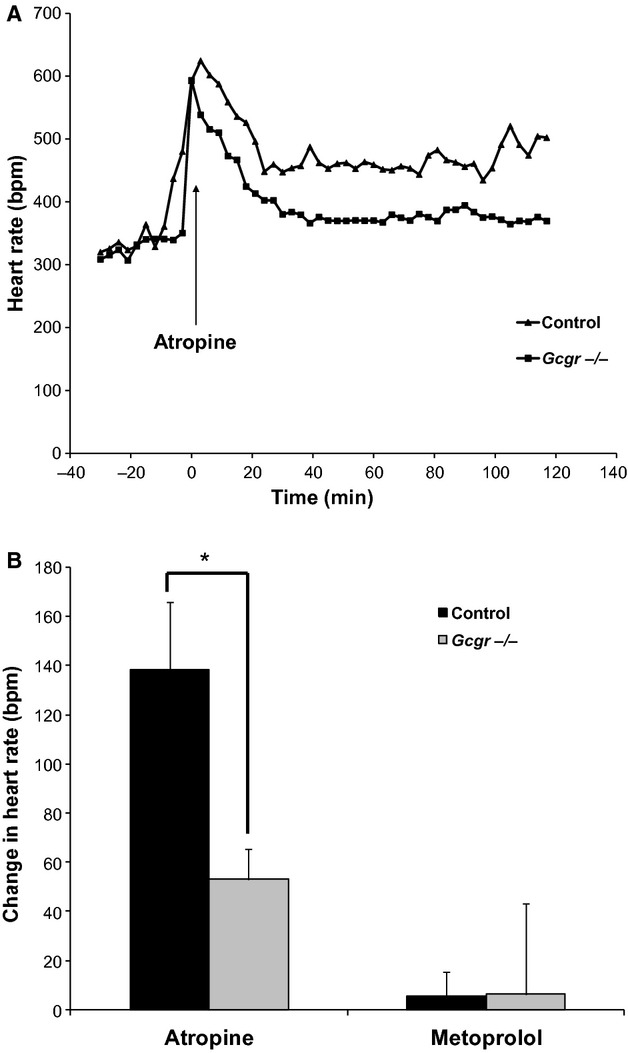
Parasympathetic activity is low in *Gcgr* −/− mice. Mice were housed at 30°C and received an intraperitoneal injection of either atropine to block PNS activity or with metoprolol to block only SNS activity. In part (A), a typical tracing from a Control mouse and a typical tracing from a *Gcgr* −/− mouse are shown in response to atropine. Tracings like those shown in part (A) were collected from all mice and the elevation in heart rate was calculated. As expected, metoprolol had no effect on HR as SNS activity is minimal at this warm *T*_a_. Atropine, however, elevated heart rate in control mice to a much greater extent than in *Gcgr* −/− mice suggesting the PNS plays a lesser role in governing heart rate in *Gcgr* −/− mice than in control mice. *P < 0.05 versus control mice.

Despite having a lower I_HR_ (Fig. 2A), *Gcgr* −/− mice have a higher resting HR than control mice (Table 2). The elevated HR in *Gcgr* −/− mice could be the result of low PNS activity at the heart and/or the result of elevated SNS activity at the heart. To distinguish between these possibilities, mice received an intraperitoneal injection of either atropine to block the muscarinic receptor or metoprolol to block the β1 adrenergic receptor. Administration of atropine affected the two groups of mice differently. When atropine was injected into control mice, HR increased by 138 ± 28 bpm (Fig. 3A and B). This demonstrates the prominent role of the PNS in depressing HR in normal mice at the warm *T*_a_ of 30ºC (Swoap et al. 2008). However, atropine elevated the HR of *Gcgr* −/− mice only 53 ± 12 bpm (Fig. 3A and B), suggesting the influence of the PNS is diminished in these mice. Metoprolol had no effect on the HR of either control or *Gcgr* −/− mice (Fig. 3B), suggesting the SNS plays little role in modulating resting HR during the light phase at 30ºC.

### Muscarinic signaling and vagal signaling are intact in *Gcgr* −/− mice

**Figure 4:**
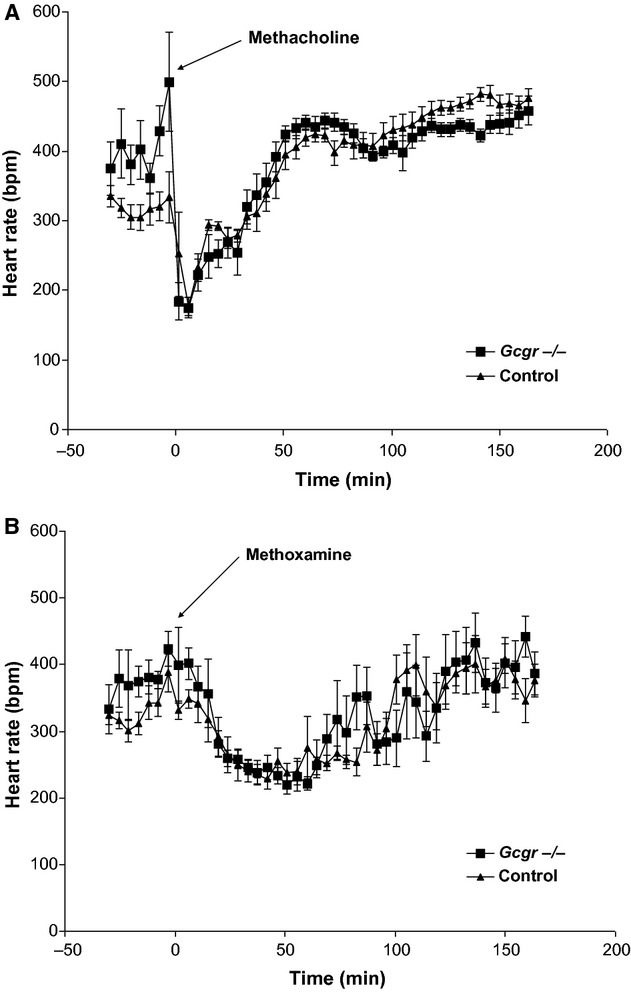
Both muscarinic receptor activity and vagal outflow to the heart are normal in *Gcgr* −/− mice. Mice were injected with either methacholine (A) or methoxamine (B) to determine where within the PNS branch is the deficit in *Gcgr* −/− mice. *Gcgr* −/− mice responded normally to methacholine, a muscarinic receptor agonist, suggesting that these mice have functioning muscarinic receptors on the heart. *Gcgr* −/− mice also responded normally to methoxamine, an α1 adrenergic receptor agonist. Methoxamine causes peripheral vasoconstriction, an elevation in blood pressure, followed by a reflex fall in heart rate that is mediated through the vagus nerve. A normal response in *Gcgr* −/− mice to methoxamine suggests that any impairment in PNS activity is upstream of the vagus nerve.

To further interrogate PNS function in *Gcgr* −/− mice, we examined the effect of methacholine and methoxamine on HR in control and *Gcgr* −/− mice. Administration of methacholine, an agonist of the muscarinic receptor, lowered HR within the first minute (Fig. 4A) in control and *Gcgr* −/− mice. Methacholine resulted in a similar time course and minimum HR for control and *Gcgr* −/− mice (138 ± 5 bpm and 132 ± 3 bpm, respectively) suggesting signaling from the muscarinic receptor is intact in *Gcgr* −/− mice. Administration of methoxamine, an agonist of the α1 adrenergic receptor, elevates blood pressure resulting in a reflex fall in HR that is mediated through the vagus nerve. Administration of methoxamine lowered HR in control mice (Fig. 4B) to 221 ± 13 bpm. The HR of *Gcgr* −/− mice fell to the same extent (201 ± 7 bpm) over the same time course as control mice (Fig. 4B), suggesting signaling through the vagus nerve is not impaired in *Gcgr* −/− mice. These results suggest the low PNS activity in *Gcgr* −/− mice is not a function of decreased vagal activity or impaired muscarinic receptor signaling.

## Discussion

As more is learned about the neuroendocrine control of hunger, and therapeutics are designed to manipulate the satiation status of individuals, it is important to consider the possible cardiac side effects of these interventions. Indeed, obesity is an independent predictor of tachycardia which is itself a strong predictor of excessive coronary morbidity and of cardiovascular mortality (Palatini et al. 1997). Examined herein are the effects of a subset of these hormones on one aspect of the cardiovascular system, I_HR_. Two anorectic hormones tested here, PYY_3-36_ and leptin, had no effect on murine I_HR_. The hunger-inducing hormone, ghrelin, similarly had no effect. CCK, which decreases hunger, has been shown to lower resting HR, with those actions mediated through the CCK-A receptor (Kurosawa et al. 2001). Thus, it was surprising to learn that CCK modestly elevates I_HR_ (Fig. 1B). To lower HR in the face of an elevated I_HR_, CCK must elevate to a great extent the vagal outflow to the heart. The last two peptide hormones tested were glucagon and OXM, both previously known to elevate I_HR_ (Steiner et al. 1969; Stuesse et al. 1982; Sowden et al. 2007). The finding that these hormones increase I_HR_ was confirmed here, with new information regarding the relative potency of these hormones on the heart (i.e., glucagon>OXM>CCK).

OXM can inhibit food intake, elevate metabolic rate, and cause weight loss when administered peripherally in humans, sparking interest in the use of OXM or related glucagon-GLP-1 receptor co-agonists as a potential therapy for obesity (Wynne et al. 2005, 2006). We and others have shown that OXM induces satiation through the GLP-1 receptor (Baggio et al. 2004; Sowden et al. 2007), so we were surprised that OXM did not require the GLP-1 receptor to elevate I_HR_ (Sowden et al. 2007). Because (1) OXM contains the identical amino acid sequence of glucagon, with an additional eight amino acids, (2) OXM has a weak affinity for the Gcgr, and (3) glucagon elevates I_HR_, we reasoned that the action of OXM on I_HR_ might be mediated through the Gcgr. To test this hypothesis, we measured the impact of glucagon and OXM on the I_HR_ in mice deficient in the Gcgr (Gelling et al. 2003). *Gcgr* −/− are viable, but have reduced plasma glucose levels, improved glucose tolerance, are resistant to diet-induced obesity, have elevated GLP-1 levels, and do not have normal fat metabolic control during a fast (Parker et al. 2002; Gelling et al. 2003; Sorensen et al. 2006; Vuguin et al. 2006; Conarello et al. 2007; Longuet et al. 2008). We show here unambiguously that neither OXM nor glucagon can modulate the I_HR_ of *Gcgr* −/− mice. These findings suggest glucagon and OXM mediate their cardiac effects through the Gcgr.

Overdoses of cardiovascular drugs are associated with morbidity and mortality. The most significant of those cardiovascular drugs are “beta-blockers” and “calcium channel blockers”. Individuals that take an overdose of these drugs include unknowing children, accidental dosing by a patient, and those trying to commit suicide. In 2004, the number of overdoses of drug-related toxic exposure to either beta-blockers or calcium channel blockers was 27,500 individuals (Watson et al. 2005). The first line of defense in an overdose of beta-blockers is glucagon injection (DeWitt and Waksman 2004; Shepard 2006) because of its inotropic and chronotropic effects on the heart independent of the beta adrenergic receptor, which is blocked in the overdose case (Kerns 2007). Glucagon is also used in combination with other antidotes in the case of an overdose of calcium channel blockers (Newton et al. 2002). However, glucagon administration in overdose cases does not consistently improve survival (Shepard 2006; Kerns 2007). Administration of glucagon to elevate HR in a beta-blocked patient can induce hyperglycemia (Taboulet et al. 1993), and so it should not be overlooked that OXM may have clinical value for these situations as OXM has antidiabetic properties associated with activating the GLP-1R (Maida et al. 2008; Pocai 2012). While glucagon is a major regulator of blood glucose, this hormone has many other effects not consistent with its counterregulatory effects to insulin (Jones et al. 2012). Alternatives to glucagon, like OXM, for positive chronotropic effects may be particularly advantageous over glucagon in humans with impaired insulin secretion (Cryer 2012; Pocai 2012). However, it should be noted that the dosage for OXM used in this study to impact murine HR is many orders of magnitude higher than that used in humans to impact satiation (Wynne et al. 2005, 2006).

While typical vivarium conditions house small rodents well below their thermoneutral zone, mice were housed in this study at 30ºC. At this *T*_a_, HR is much lower than I_HR_ and much lower than when mice are housed at cooler *T*_a_s (Williams et al. 2002; Swoap et al. 2008). In wild-type mice, the bradycardia with exposure to a warm environment is a result of an increased cardiac influence of the PNS via the vagus nerve and decreased influence of the SNS. Two pieces of evidence suggest that PNS activity to the *Gcgr* −/− mouse heart is diminished: (1) the elevated HR in the light phase in *Gcgr* −/− mice at 30ºC (Table 2), when PNS activity is at its greatest, and (2) the only modest elevation in HR response to atropine (Fig. 3A and B), an antagonist of the muscarinic receptor on the heart. Many potential levels along the PNS chain could be responsible for the lack of cardiac suppression in *Gcgr* −/− mice housed near their thermoneutral zone. These levels include acetylcholine insensitivity, lack of acetylcholine release, some other defect in the vagus nerve itself, generation of the initial action potentials within the brainstem, or sensory inputs into the PNS center. We show here that signaling through the vagus nerve appears normal because *Gcgr* −/− mice responded normally to methoxamine (Fig. 4). This compound is a potent vasoconstrictor that elevates blood pressure and induces a reflex fall in HR via the vagus nerve. Similarly, the signaling at the heart through the muscarinic receptor seems normal because *Gcgr* −/− mice responded normally to methacholine, a muscarinic receptor agonist (Fig. 4). It remains to be determined whether afferent nerve activity reporting temperature (external or internal) is somehow impaired in *Gcgr* −/− mice, or some defect further downstream from temperature sensation. However, our data suggest that activity from the vagus nerve and activity at the cardiac muscarinic receptor are not impaired.

To summarize, OXM is a potent appetite suppressant. Its satiation activity is mediated through the GLP-1R (Cohen et al. 2003; Dakin et al. 2004; Sowden et al. 2007). OXM also robustly elevates the I_HR_ of a mouse. Although the majority of glucagoregulatory actions of OXM are mediated through the GLP-1R (Maida et al. 2008), we show here that the cardiac effects of OXM (and glucagon) are mediated through the Gcgr. It is also suggested that OXM may be an excellent alternative to glucagon for the therapeutic treatment of acute overdose of beta-blockers.
